# Organizational Context and Quality Indicators in Nursing Homes: A Microsystem Look

**DOI:** 10.1177/07334648231200110

**Published:** 2023-09-05

**Authors:** Yinfei Duan, Matthias Hoben, Yuting Song, Stephanie A. Chamberlain, Alba Iaconi, Katharina Choroschun, Shovana Shrestha, Greta G. Cummings, Peter G. Norton, Carole A. Estabrooks

**Affiliations:** 1 Faculty of Nursing, 3158 University of Alberta, Edmonton, AB, Canada; 2 Faculty of Health, 56014 York University, Toronto, ON, Canada; 3 School of Nursing, 12593 Qingdao University, Qingdao, China; 4 School of Public Health, Bielefeld University, Bielefeld, Germany; 5 Department of Family Medicine, 2129 University of Calgary, Calgary, AB, Canada

**Keywords:** organizational context, work environment, nursing home quality, quality indicators, clinical microsystem

## Abstract

The association of organizational context with quality of care in nursing homes is not well understood at the clinical microsystem (care unit) level. This cross-sectional study examined the associations of unit-level context with 10 unit-level quality indicators derived from the Minimum Data Set 2.0. Study settings comprised 262 care units within 91 Canadian nursing homes. We assessed context using unit-aggregated care-aide-reported scores on the 10 scales of the Alberta Context Tool. Mixed-effects regression analysis showed that structural resources were negatively associated with antipsychotics use (B = −.06; *p* = .001) and worsened late-loss activities of daily living (B = −.03, *p* = .04). Organizational slack in time was negatively associated with worsened pain (B = −.04, *p* = .01). Social capital was positively associated with delirium symptoms (B = .12, *p* = .02) and worsened depressive symptoms (B = .10, *p* = .01). The findings suggested that targeting interventions to modifiable contextual elements and unit-level quality improvement will be promising.

**What this paper adds**

• The current study was an initial attempt to assess organizational context and quality of care with clinical microsystem (care unit) being the focal point of investigation.
• We observed that 3 elements of organizational context at the clinical microsystem level contributed to cross-unit variations in quality indicators: care aides’ accessibility to structural resources, perceived slack of time, and social capital.

**Applications of study findings**

• The findings of this study indicate the potential use of the clinical microsystem (care unit) in health serveries research and quality improvement activities in the nursing home context.
• Future studies can build on these findings to further investigate the mechanisms by which context affects quality of care at the clinical microsystem level.
• Targeting contextual elements at the clinical microsystem level and the use of unit-level quality outcome measures will be promising to implement and evaluate quality improvement interventions in nursing homes.

## Introduction

An aging population with a growing prevalence of dementia and other chronic conditions contributes to an increase in demand for care in nursing homes (Estabrooks et al., 2020). However, poor quality of care in nursing homes is a widespread concern in Canada, and around the world (Estabrooks et al., 2020). Approximately 225,000 older Canadians live in around 2076 nursing homes or long-term care homes, which provide 24/7 supervised care akin to the skilled nursing facilities in the U.S. (Canadian Institute for Health Information, 2021a) Nursing home care in Canada remains outside universally insured health services protected by the Canada Health Act (Estabrooks et al., 2020). Instead, it falls under the jurisdiction of provincial and territorial governments, leading to a significant variation in funding, service provision, and regulation across the country (Armstrong et al., 2020; Estabrooks et al., 2020). Additionally, the Canadian nursing home sector features a diverse mix of public, non-profit, and for-profit providers (Canadian Institute for Health Information, 2021a). Similar to other countries, challenges to providing optimal care in Canadian nursing homes include increasing complexity in care needs (Canadian Institute for Health Information, 2021c), limited availability of appropriate treatment and care (Canadian Institute for Health Information, 2021b; Hoben et al., 2016; Perlman et al., 2019), and a shortage of staffing and skill mix (Chamberlain et al., 2019; Duan et al., 2020; Song et al., 2020).

Quality of care varies across nursing homes, with organizational context a major contributing factor to these differences (Backhaus et al., 2014; Baldwin et al., 2017). Organizational context is defined in health care as the environment or setting in which people receive health care services, where research evidence is translated into practice, and quality improvement interventions are implemented (McCormack et al., 2002). Organizational context comprises structural (e.g., ownership models, resources), cultural (e.g., norms, values), and social (e.g., communications, network) elements, of which some are modifiable (McCormack et al., 2002).

Studies examining the impacts of organizational context on the quality of nursing home care have primarily focused on non-modifiable structural factors such as facility size and ownership (Baldwin et al., 2017; Harrington et al., 2001; McGregor et al., 2006; O’Neill et al., 2003). The results, although important, have provided limited practical guidance to design quality improvement interventions. Other studies investigating modifiable structural factors, such as staffing and skill mix, have offered mixed results (Backhaus et al., 2014). Despite emerging research to examine the relationship between non-structural factors of organizational context (such as managerial practices, leadership, and communication) and quality of care outcomes in nursing homes (Aiken et al., 2002; Anderson et al., 2003; Barry et al., 2005; Flynn et al., 2010; Temkin-Greener et al., 2009, 2010, 2012; White et al., 2020), knowledge gaps remain—as summarized below.

Studies have measured organizational context as reported by administrators, managers, or regulated staff such as registered nurses (Anderson et al., 2003; Barry et al., 2005; Flynn et al., 2010; White et al., 2020), and usually exclude care aides (unregulated staff). Care aides are primary caregivers who provide full-time direct care to nursing home residents (Berta et al., 2013; Hewko et al., 2015). Organizational context as perceived by care aides can offer insights into how care is delivered, how people interact locally, and how proposed change is implemented at the point of care (Janes et al., 2008; Madden et al., 2017). As the call for transformative or cultural change in nursing home care continues to grow—changes that emphasize empowerment of residents, frontline care staff, and family caregivers—the voices of care aides have become increasingly vital in both the practice and research within this sector (Koren, 2010). The implementation of person-centered care is being significantly influenced by empowering care aides and including their voices (Duan et al., 2021; Grabowski et al., 2014, 2016; Hamann, 2014), as exemplified by innovative culture change models like the Green House model, where care aides, referred to as Shahbazim, form a self-managed care team and collectively transform their work environment to be more person-centered and relationship-centered, resulting in improved quality of care outcomes (Loe & Moore, 2012; Zimmerman et al., 2016). However, the broad inclusion of care aides’ perspectives and insights in health services and organizational research has yet to be widely adopted, particularly quantitative survey studies (Gaugler, 2014).

Further studies have often examined contextual factors and quality of care outcomes at the facility level, whereas contextual factors at the care unit level have been rare. A care unit represents a clinical microsystem in which a small group of people work together on a regular basis to provide care to discrete subpopulations of patients (Nelson et al., 2002). A care unit shares aims, processes, information, and outcomes. Reported variations in quality of care across units within the same nursing home demonstrate that care units are increasingly recognized as a focus for health services research and quality improvement practice (Estabrooks et al., 2011a; Lanham, 2013; Nakrem, 2015; Norton et al., 2014).

The purpose of this study is to systematically identify organizational context factors that are associated with quality indicators (QIs) at the clinical microsystem level in nursing home settings. Specifically, we examined modifiable traits of organizational context measured using the Alberta Context Tool (reported by care aides and aggregated to the unit level) and their associations with 10 unit-level QIs derived from the Resident Assessment Instrument-Minimum Data Set 2.0 (RAI-MDS 2.0).

## Methods

### Study Design

This was a retrospective cross-sectional study of data collected from the Translating Research in Elder Care (TREC) program. TREC is an applied health services research program that focuses on improving quality of care and quality of life for residents, and quality of work-life for care staff in nursing homes in Canada (Estabrooks et al., 2009). This study is part of a parent study: the Influence of Context on Implementation and Improvement (ICII) (Estabrooks et al., 2022). The ICII project aims to comprehensively analyze data from TREC’s multiple data sources. Its goal is to investigate the relationships of context with implementation success, staff quality of work-life outcomes, and resident quality of care outcomes. TREC has survey data collected since 2008 from both regulated (i.e., nurses, physicians, applied professionals, and care managers) and unregulated staff (i.e., care aides) working in nursing homes in 4 Canadian provinces. Access to RAI-MDS 2.0 data from participating nursing homes is also available. In this study, we used TREC data collected from September 2019 to the first week of March 2020, a timeframe preceding the first wave of the COVID-19 outbreak in Canada (Urrutia et al., 2021). These data were gathered through a care aide survey, a unit survey, a facility survey, and RAI-MDS 2.0.

### Setting and Participants

The study setting included 91 nursing homes across 3 Canadian provinces: Alberta, British Columbia, and Manitoba. Nursing homes were selected using stratified random sampling, with strata comprising region, facility owner-operator model, and facility size (Estabrooks et al., 2009). Data were collected from the following groups of participants: (1) care aides (*n* = 3765) who completed the care aide survey, (2) facility administrators or directors of care (*n* = 91) who completed the facility survey, (3) unit managers (*n* = 324) who completed the unit survey, and (4) residents (*n* = 12 414) who lived at the participating nursing homes during TREC data collection, as well as had available RAI-MDS 2.0 data. The data of the care aide survey, facility survey, and unit survey were collected through in-person structured interviews conducted by on-site TREC regional coordinators with their respective participants (Estabrooks et al., 2009).

Through TREC, we have access to identifiers of care units in both the survey dataset and the RAI-MDS 2.0. To link the different sources of data, we aggregated care aide survey data to the unit level and calculated unit-level RAI-MDS 2.0 QIs, then linked these data with the TREC facility and unit survey data. Detailed information about sampling and recruitment methods has been published elsewhere (Estabrooks et al., 2009).

Additional publications have validated and reported on the operational definition of a care unit as a representation of a clinical microsystem (Estabrooks et al., 2011a; Norton et al., 2014). A care unit is defined as a geographical area in a facility, serving a population of residents, and is characterized by, (1) a regular group of care providers (e.g., care aides, licensed practical nurses, and registered nurses) who work most of their shifts (typically at least 60%) on one unit, and (2) a care manager who is in charge of the whole unit plus a nurse who oversees the unit on a shift-by-shift basis (though the supervision of a care manager or a charge nurse may be spread across several units) (Estabrooks et al., 2011a; Norton et al., 2014).

The analytic sample comprised 262 care units within the participating 91 nursing homes. We excluded 39 care units (of the original 324 care units) that had less than 8 care aide respondents to ensure stable unit-aggregated Alberta Context Tool (ACT) scores (Estabrooks et al., 2011b). We also excluded 23 care units with outliers in unit covariates including total nursing staff hours and percentage of care aide hours. Care units that were excluded from the analysis were more likely to be from private for-profit homes, or homes with a fewer number of services or care programs. The only significant difference found in QIs between care units that were excluded and included from the analysis was the higher prevalence of physical restraint use in the excluded units.

### Variables and Data Sources

#### Outcome Variables

Outcomes included 10 QIs derived from RAI-MDS 2.0. A QI is represented as the proportion of residents who present with a certain condition at a single point in time (a prevalence indicator) or the proportion of residents with a new presentation of a certain condition that compares 2 points in time (an incidence indicator) (Canadian Institute for Health Information, 2020; Estabrooks et al., 2013). Among 35 RAI-MDS 2.0 QIs endorsed by the Canadian Institute for Health Information (CIHI) (Canadian Institute for Health Information, 2020), we selected 10 that have been previously identified as sensitive to care practices in nursing homes (Estabrooks et al., 2013). Table 1 provides further description.

**Table 1.** Description of Unit-Level Quality Indicators, Context Variables, and Control Variables.

Variables	Description
*Unit-level quality indicators* obtained from Resident Assessment Instrument-Minimum Data Set 2.0	
RES01	% of residents in daily physical restraints
PRU05	% of residents who had stage 2–4 pressure ulcers
WGT01	% of residents who had unexplained weight loss
PAN01	% of residents whose pain worsened
BEHD4	% of residents whose behavioral symptoms worsened
DEL0X	% of residents with symptoms of delirium
ADL01	% of residents with whose late-loss ADL functioning worsened
FAL02	% of residents who fell in the last 30 days
MOD4A	% of residents whose depressive symptoms worsened
DRG01	% of residents on antipsychotics without a diagnosis of psychosis

*The Alberta Context Tool reported by care aides *obtained from the TREC care aide survey		
Leadership	The actions of formal leaders on the unit to influence practice, 6 items (e.g., “the person I report to looks for feedback even when it is difficult to hear”).	Each item was measured on a 5-level scale (strongly disagree-strongly agree, range = 1–5).
Culture	The way that “we do things” on the unit, reflecting supportive work culture, 6 items (e.g., “I receive recognition about my work”).	
Evaluation	The process of using data to assess team performances and to achieve outcomes, 6 items (e.g., “I routinely receive information on my team’s performance”).	For each scale, a composite score was obtained by taking the mean of the items making up the scale.
Social capital	Active connections among people (bonding, bridging, and linking), 6 items (e.g., “people on the team share information with others”).	
Organizational slack (OS)	The cushion of actual or potential resources which allows for adapting to internal pressures:	
Staffing	3 items (e.g., we have enough staff to deliver best possible care).	
Space	2 items (e.g., we have private space such as a conference room to discuss resident care).	
Time	4 items (e.g., we have time to do something extra for residents).	
Formal interactions	Formal exchanges between care aides and other staff through scheduled activities, 4 items (e.g., team meetings, change of shirt report).	Each item was original measured on 5-level scale (1 = never to 5 = almost always) and recoded 1 and 2 to 0, 3 to .5, 4 and 5 to 1. Sum recoded items to obtain a scale score.
Informal interactions	Information exchanges between individuals, 9 items (e.g., hallway talk, informal bedside teaching).	
Structural resources	The structural elements of an organization that facilitate the use of knowledge, measured with 7 items (e.g., notice boards, journals in the work area, policies and procedure manuals).	
*Facility characteristics* obtained from TREC facility survey (completed by facility administrators or director of care through in-person structured interviews)		
Facility size	Small (<80 beds), medium (80–120 beds), large (>120 beds)	
Ownership model	Public not for profit, private for profit, voluntary not for profit	
Services or care programs	A count of 17 services or care programs that are available in the facility, for example, physiotherapist, social worker, recreational therapist, and dietician.	
Quality of improvement activities	A count of 5 quality of improvement activities carried out in the facility, for example, regular use of data for quality improvement, education sessions about quality improvement.	
*Unit characteristics* obtained from the TREC unit survey (completed by unit managers through in-person structured interviews)		
Unit type	Secure dementia, non-secure dementia, general long-term care unit, other types	
Total care hours	Care hours per resident day provided by registered nurses, licensed practical nurse, and care aides (this variable was derived using the manager-reported number of staff in each category).	
Ratio of care hours by care aides	Care hours per resident day provided by care aides divided by total care hours provided by registered nurses, licensed practical nurse, and care aides. (Care hours per resident day provided by care aides was derived using the manager-reported number of care aides)	

Although QIs are generally measured at the facility level, we calculated them at the unit level (Norton et al., 2014). Specifically, we used unit-level risk-adjusted QIs reported for the quarter immediately following the period in which the TREC survey data collection was completed for that care unit. This would ensure temporal congruence among variables such that the outcome data, namely, QIs, succeeded the measurement of context variables.

Unit-level QIs were risk-adjusted following the technical guide developed by the CIHI, which combines, specific to each QI, (1) clear inclusion/exclusion criteria for both the numerator (residents with the condition associated with a certain QI) and the denominator (residents with valid assessment on the care unit); (2) stratification of the sample by highly influential resident characteristics (e.g., cognitive or physical function); and (3) regression-based adjustment for additional covariates (e.g., age and case mix index) (Canadian Institute for Health Information, 2013, 2020). The risk adjustment processes took into account differences in the risk profiles of resident populations within individual care units to enable between-unit comparison. The risk adjustment methods are detailed in the Supplemental File S1 and Supplemental Table S1.

#### Explanatory Variables

In the care aide survey, the ACT was used to measure microsystem-level organizational context as perceived by care aides (Estabrooks et al., 2011b). The 10 scales of the ACT reflect 10 domains of organizational context, including leadership, culture, evaluation (feedback), formal and informal interactions, structural and electronic resources, social capital, and organizational slack in time/staffing/space (Table 1) (Estabrooks et al., 2011b).

We aggregated each ACT scale to the unit level by taking the average of scale scores or counts reported by care aide respondents on the unit. The reliability (i.e., internal consistency) and validity (i.e., construct and predictive validity) of the ACT scales have been reported elsewhere (Estabrooks et al., 2011b). For each scale, the reliability of the aggregated score was tested against various common aggregation indices and the results indicated the reliability of aggregating individual responses using unit mean when there are 8 or more care aides respondents on the unit (Estabrooks et al., 2011b).

#### Control Variables

We included facility and unit covariates (Table 1) in regression models. These variables represent facility- and unit-level structural characteristics that are associated with quality outcomes in nursing homes, or have a theoretical influence on quality of care (Backhaus et al., 2014; Baldwin et al., 2017; O'Neill et al., 2003; Zinn et al., 2003). Facility-level structural characteristics, obtained from the facility survey, included owner-operator model, facility size, services/care programs available in the facility, and quality improvement activities carried out in the facility. Unit-level structural characteristics (i.e., staffing variables), obtained from the unit survey, included total nursing staff hours per resident day and percentage of care aide hours. The staffing variables were derived using the manager-reported number of staff (Cummings et al., 2017).

### Statistical Analysis

We calculated descriptive statistics (mean, median, and standard deviation) for each QI, context variable, and other characteristics of the unit sample. We used Pearson’s correlation to analyze correlation of each QI with individual ACT scales. A series of mixed-effects linear regressions were conducted to examine the associations between organizational context and QIs while controlling for covariates. Specifically, we used 2-level mixed-effect linear regression to control for the clustering of care units nested within the same facility. We included all ACT scales collectively in each regression model, except for that of evaluation and organizational slack in staffing. These 2 scales were highly correlated with other ACT scales and including them would result in multicollinearity (Pearson’s correlation of evaluation with other ACT scales had a range of .17–.59; Pearson’s correlation of organizational slack in staffing with other ACT scales had a range of .21–.67). All analyses were conducted using Stata 16 (StataCorp, 2019).

## Results

Among 262 care units included in the current analysis, about 43% of care units were within private for-profit nursing homes and over half (58%) were within large nursing homes (>120 beds) (Table 2). Over two-thirds of care units (73%) were general long-term units and 15% were secure dementia units. Table 2 provides descriptive statistics of the unit-level ACT scores. Descriptive statistics of the 10 QIs are supplied in Table 3.

**Table 2.** Descriptive Statistics of Context Variables and Control Variables for the Analytic Sample of Care Units (*n* = 262).

Variable (Potential range for continuous variables)	Mean	SD	Min	Max
The Alberta context tool scales				
Leadership (1–5)	4.0	0.2	3.2	4.5
Culture (1–5)	4.1	0.2	3.5	4.6
Evaluation (1–5)	3.8	0.2	2.9	4.3
Social capital (1–5)	4.0	0.2	3.4	4.6
Formal interactions (0–4)	1.5	0.3	0.7	2.3
Informal interactions (0–9)	4.1	0.6	2.4	5.8
Structural resources (0–7)	2.6	0.7	0.6	4.3
Organizational slack-staffing (1–5)	2.8	0.6	1.5	4.1
Organizational slack-space (1–5)	3.5	0.7	1.5	4.8
Organizational slack-time (1–5)	3.5	0.4	2.2	4.3

Control variables	Freq	%
Region		
Edmonton zone	60	22.9
Calgary zone	54	20.6
BC Interior health	22	8.4
BC Fraser health	77	29.4
Winnipeg regional health authority	49	18.7
Facility owner-operator model		
Public not for profit	42	16.0
Private for profit	113	43.1
Voluntary not for profit	107	40.1
Facility size		
Small (<80 beds)	32	12.2
Medium (80–120 beds)	78	29.8
Large (>120 beds)	152	58.0

	Mean	SD	Min	Max
Services/care programs available in the facility (0–17)	7.5	2.0	2.0	14.0
Quality of improvement activities carried out in the facility (0–5)	3.9	1.0	0.0	5.0
Total care hours per resident day (>=0)	2.7	0.6	1.3	4.5
Ratio of care hours per resident day provided by care aides (0–1)	0.7	0.1	0.4	0.9

*Note.* BC: British Columbia.

**Table 3.** Descriptive Statistics of the Risk-Adjusted, Unit-Level Quality Indicators for the Analytic Sample of Care Units (*n* = 262).

Quality indicators (Description)	Descriptive statistics					
	Mean	SD	Minimum	Median	Maximum	IQR
RES01 (Daily physical restraints)	.03	.05	0	.00	.35	.04
PRU05 (Stages 2–4 pressure ulcers)	.04	.06	0	.01	.31	.06
WGT01 (Unexplained weight loss)	.08	.09	0	.07	.59	.12
PAN01 (Worsened pain)	.09	.08	0	.08	.37	.12
BEHD4 (Worsened behavioral symptoms)	.13	.09	0	.11	.46	.11
DEL0X (Symptoms of delirium)	.14	.13	0	.12	.63	.20
ADL01 (Worsened late-loss ADL)	.16	.12	0	.14	.58	.16
FAL02 (Fell in the last 30 days)	.16	.11	0	.14	.65	.15
MOD4A (Worsened depressive symptoms)	.18	.13	0	.16	.54	.19
DRG01 (Antipsychotics)	.20	.14	0	.17	.97	.16

*Note.* Each quality indicator is presented as the proportion of residents with a given condition on the care unit. IQR: interquartile range.

Pearson’s correlation shows that all of the examined QIs (except for the prevalence of daily use of physical restraints and the incidence of worsened behavioral symptoms) were significantly correlated with at least one ACT scale (Table 4). However, the strength of correlations was small (value <.3). By contrast, not all ACT scales, such as those of culture and evaluation, correlated with QIs. For the scales of leadership, formal interactions, organizational slack in staffing, and organizational slack in time, higher ACT scores were associated with lower QIs (i.e., a lower prevalence or incidence of certain clinical conditions). However, higher scores on the social capital scale and the scale of informal interactions were associated with higher QIs and the scales of structural resources and organizational slack in space showed mixed results.

**Table 4.** Pearson Correlations Between Quality Indicators and Alberta Context Tool (ACT) Scales.

	RES01 (Physical restraints)	PRU05 (Pressure ulcers)	WGT01 (Weight loss)	PAN01 (Worsened pain)	BEHD4 (Worsened behavioral symptoms)
Leadership	.03	.09	−.04	−.01	.09
Culture	.01	.04	−.01	.08	.10
Evaluation	.02	.04	−.05	.09	.07
Social capital	.04	.12	.09	.04	.12
Formal interactions	.00	.08	.02	.07	.05
Informal interactions	−.02	.13*	.08	.07	.06
Structural resources	.04	.13*	−.12*	.09	.04
OS-staffing	.08	.02	−.09	−.09	−.06
OS-space	.09	.08	−.11	.14*	.02
OS-time	.03	.01	−.02	−.08	−.04

	DEL0X (Symptoms of delirium)	ADL01 (Worsened late-loss ADL)	FAL02 (Fall)	MOD4A (Worsened depressive symptoms)	DRG01 (Antipsychotics)
Leadership	.02	−.17*	.03	.02	−.02
Culture	.05	−.10	.03	.07	.00
Evaluation	−.02	−.07	−.02	−.03	−.02
Social capital	.20*	.03	.08	.18*	−.02
Formal interactions	.02	−.10	−.15*	.04	.00
Informal interactions	.03	−.05	−.06	.06	.01
Structural resources	.02	−.25*	−.13*	.03	−.17*
OS-staffing	−.09	−.11	.01	−.13*	−.04
OS-space	.24*	−.16*	−.03	.19*	−.15*
OS-time	−.08	−.19*	−.03	−.09	−.03

*Note.* OS: Organizational slack. *p < .05.

Regression analysis shows that none of the ACT scales were significantly associated with the following QIs: the use of physical restraints, pressure ulcers, unexplained weight loss, worsened behavioral symptoms, and falls (Table 5). Higher scores on the ACT scale of structural resources were significantly associated with a lower incidence of late-loss activities of daily living (B = −.03; 95% CI: −.06, −.001; *p* = .04) and a lower prevalence of antipsychotic use (B = −.06; 95% CI: −.10, −.02; *p* = .001). Higher scores on the ACT scale of organizational slack in time were significantly associated with a lower incidence of worsened pain (B = −.04; 95% CI: −.07, −.01; *p* = .01). However, higher scores on the social capital scale were significantly associated with a higher prevalence of delirium symptoms (B = .12; 95% CI: .03, .21; *p* = .02) and a higher incidence of worsened depressive symptoms (B = .10; 95% CI: .02, .18; *p* = .01).

**Table 5.** Associations Between Quality Indicators (QIs) and Alberta Context Tool (ACT) Scales: Results (Regression Coefficients and 95% CI) From 2-Level Random Intercept Linear Regression.

	RES01 (Daily physical restraints)	PRU05 (Stage 2–4 pressure ulcers)	WGT01 (Unexplained weight loss)	PAN01 (Worsened pain)	BEHD4 (Worsened behavioral symptoms)
The Alberta context tool scales					
Leadership	.00 (−.03, .04)	.03 (−.01, .07)	−.01 (−.07, .05)	.00 (−.05, .05)	.05 (−.01, .11)
Culture	−.01 (−.05, .03)	−.02 (−.07, .03)	.01 (−.06, .08)	.04 (−.02, .1)	.03 (−.04, .1)
Social capital	.01 (−.03, .05)	.03 (−.02, .08)	.06 (0, .13)	−.01 (−.07, .05)	.03 (−.04, .1)
Formal interactions	.01 (−.01, .04)	−.01 (−.04, .02)	.00 (−.05, .04)	−.01 (−.05, .03)	.00 (−.04, .05)
Informal interactions	.00 (−.01, .01)	.01 (0, .03)	.00 (−.02, .02)	.01 (−.01, .02)	.00 (−.02, .02)
Structural resources	.01 (−.01, .02)	.01 (−.01, .02)	−.01 (−.03, .01)	.00 (−.02, .02)	.00 (−.03, .02)
OS-space	.00 (−.02, .01)	.00 (−.02, .01)	−.01 (−.03, .01)	.00 (−.01, .02)	−.01 (−.03, .01)
OS-time	.00 (−.03, .02)	−.02 (−.05, .01)	.01 (−.03, .06)	−.04* (-.07, .01)	−.03 (−.07, .01)
Intercept	.03 (−.16, .21)	−.16 (−.38, .06)	−.25 (−.58, .07)	.10 (−.18, .37)	−.24 (−.57, .08)
ICC	.42 (.29, .56)	.03 (.00, .43)	.40 (.26, .56)	.19 (.10, .35)	.04 (.00, .43)
R^2^	.04	.08	.04	.13	.07
*n*	260	259	257	260	260

	DEL0X (Symptoms of delirium	ADL01 (Worsened late-loss ADL)	FAL02 (Fall in the last 30 days	MOD4A (Worsened depressive symptoms)	DRG01 (Antipsychotics)
The Alberta context tool scales					
Leadership	.02 (−.07, .10)	−.04 (−.12, .04)	.04 (−.03, .12)	.01 (−.06, .09)	.03 (−.07, .13)
Culture	−.03 (−.12, .07)	.03 (−.06, .12)	.04 (−.05, .13)	.05 (−.04, .13)	.01 (−.10, .13)
Social capital	.12* (.03, .21)	.07 (−.02, .15)	.03 (−.06, .11)	.10* (.02, .18)	.02 (−.09, .13)
Formal interactions	−.03 (−.09, .03)	.00 (−.06, .05)	−.04 (−.09, .02)	−.03 (−.08, .03)	.05 (−.03, .12)
Informal interactions	.01 (−.01, .04)	.00 (−.03, .02)	−.01 (−.04, .01)	.00 (−.02, .03)	.00 (−.04, .03)
Structural resources	.00 (−.03, .04)	−.03* (-.06, -.01)	−.01 (−.04, .02)	−.01 (−.04, .02)	−.06** (-.10, -.02)
OS-space	.02 (−.01, .04)	−.01 (−.04, .01)	−.01 (−.03, .01)	.01 (−.02, .03)	−.02 (−.05, .01)
OS-time	−.02 (−.08, .04)	−.02 (−.07, .04)	.00 (−.05, .05)	−.03 (−.08, .03)	.04 (−.03, .11)
Intercept	−.34 (−.77, .09)	.05 (−.36, .45)	−.25 (−.64, .14)	−.51* (−.91, −.11)	−.12 (−.64, .39)
ICC	.35 (.23, .50)	.19 (.08, .38)	.17 (.07, .34)	.48 (.34, .61)	.11 (.03, .30)
R^2^	.06	.08	.07	.07	.08
*n*	255	256	259	259	256

*Note.* For each QI, we used 2-level mixed-effect linear regression to examine the relationship of the QI with eight of the Alberta Context Tool scales, controlling for the clustering of care units nested within the same facility. We also controlled for covariates including facility size, ownership model, services/care programs, quality of improvement activities, total care hours per resident day, and percent of care hours per resident day provided by care aides. Marginal R^2^ was calculated, which focuses on variance of the outcome explained by fixed factors (Nakagawa & Schielzeth, 2013). We used Variance_null_-Variance_model_/Variance_null_ to calculate marginal R^2^. Variance_model_ is the residual variance of the full model that includes fixed effects of predictors and the random effect. Variance_null_ is the residual variance of the null model that includes the intercept and random effect only with the random effect being constrained to be the same as that in the full model. Coefficients and 95% CIs of the covariates are presented in the Supplemental Table S2. *p < .05; **p < .01; ICC = Intra-class correlation; OS: Organizational slack.

The intra-class correlations (ICCs) varied substantially across the unit-level QIs. QIs related to the use of physical restraints, unexplained weight loss, delirium symptoms, and worsened depressive symptoms had ICCs between .35 and .48, indicating that over a third to nearly half of the total variability in these QIs was due to the variability between facilities, as opposed to the variability within facilities. Conversely, QIs related to pressure ulcers and worsened behavioral symptoms had ICCs <.05, indicating that minimal variability in these two QI was due to between-facility variability. QIs related to worsened pain, worsened late-loss activities of daily living, falls, and antipsychotic use had ICCs between .10 and .20. Based on the marginal R^2^, which focuses on the variance of the outcome attributed to fixed factors, the models account for 4%–13% of the variance in the QIs.

## Discussion

This study examined the relationships between organizational context (modifiable elements of work environment) and QIs at the clinical microsystem level. The findings suggest that access to various structural resources for care aides and their perceptions of having sufficient time to provide optimal resident care (organization slack in time) were associated with better clinical outcomes. This was most apparent in areas of resident care related to the use of antipsychotics, activities of daily life, or pain. Unexpectedly, more social capital (a reflection of connectedness among people in a team) was associated with worsened depressive symptoms and a higher prevalence of delirium symptoms. Standard errors (confidence intervals) for some of the associations were large, suggesting wide variation of these associations across units.

Structural and information resources (such as clinical guidelines, textbooks, and in-services) that are readily available for care aides can be vital in maintaining and improving quality of care in the nursing home setting. Previous studies have explored how the use of those resources by care aides can affect quality of care. Using the ACT measure of structural resources, Estabrooks et al. (2015) reported that frequent use of structural resources by care aides in nursing homes was positively associated with using evidence-based knowledge and best practices in decision-making processes (e.g., increasing awareness around new ways to care for residents). A systemic review of implementation literature across health care settings also identified that consistent use of educational materials by staff influenced successful evidence-based practices (Li et al., 2018). The changing perceptions of care aides and implementation of evidence-based practices through structural and information resources may further lead to improved quality of care outcomes. Nevertheless, more research is needed to uncover the causal links between structural resources and specific quality outcomes (such as reducing the use of antipsychotics and maintaining activities of daily life).

More available time (organization slack in time), as perceived by care aides, was associated with a lower proportion of residents with worsened pain and is in line with findings from previous studies. Care aides play a major part in pain management processes, particularly in monitoring pain and facilitating nonpharmacological interventions (Liu, 2014). Other studies have reported time constraints as barriers to completing these processes (Brunkert et al., 2020; Weiner & Rudy, 2002). Interestingly, the significant association between organization slack in time and the pain QI persisted after we controlled for the objective measures of staffing (i.e., nursing care hours per resident day and percentage of care hours provided by care aides). This finding highlights the importance of measuring time resources from the viewpoint of care aides as a complement to objective measures of staffing. Perceived time buffering or time constraints are not associated solely with objective measures of staffing resources, but also a reflection of teamwork, connectivity and collegiality among staff, effective information sharing, and efficient work procedures (Forbes-Thompson et al., 2007; Janes et al., 2008). Future research should focus on the relationship of perceived time resources with care quality and how subjective measures of time resources differentiate from objective measures of staffing in predicting quality outcomes.

In contrast to our expectations, we observed that a 1-point increase in the score of the social capital scale was associated with a more than 10% increase in the proportion of residents with delirium symptoms or worsened depressive symptoms. In this study, social capital captured the extent to which care aides could freely exchange information about resident care and support each other in the job. It was unclear why reports of higher levels of social capital were associated with a higher prevalence of delirium symptoms or worsened depressive symptoms. One speculation is that good teamwork might lead to effective detection and communication of residents’ symptoms such as those of delirium or depressive mood, whereas members in care teams with poor social capital may be reluctant to communicate the detection of these symptoms due to unfavorable working relationships, or fears of being blamed for poor outcomes, leaving these symptoms under documented. Previous studies have consistently suggested that poor working relationships among care aides, or between care aides and nurses, could lead to breakdown in the transfer of important information about the health conditions and care needs of residents (Anderson et al., 2003; Temkin-Greener et al., 2010, 2012).

Another speculation is that a high prevalence of delirium symptoms or worsened depressive symptoms on a care unit might lead to more reliance on strong teamwork such that social capital of the care team would be enhanced (Caspar et al., 2016; Janes et al., 2008; Madden et al., 2017; Yoon et al., 2020). According to a grounded theory study by Janes et al. (2008) teamwork and positive relational conditions among staff are critical for care aides to apply dementia care practice where unpredictability, variability, and uncertainty are inherent. It is common for residents with dementia to experience symptoms of delirium (e.g., acute confusion and disturbance in attention), changeable moods, and worsening depressive symptoms, which can produce an inconsistent and unpredictable work environment (Borza et al., 2022; Cheung et al., 2018). It remains important for future research to examine the causal relationship between social capital and quality outcomes. An intervention study that targets fostering social capital will help determine if coherent and collaborative work between care aides and the care team improves outcomes for managing depressive symptoms and delirium.

### Implications

Our study provides a preliminary examination of the links between unit-level context and QIs; future studies can build on these findings and further investigate the mechanisms by which context affects QIs at the unit level. Potential pathways linking organizational context and the quality of care may include staff behaviors of implementing evidence-based practices, implementation of quality initiatives or clinical interventions, organizational learning behaviors, and work-life-related outcomes for staff.

Our research underscored the value of considering care aides’ perceptions of their immediate work environment within a care unit, referred to as the clinical microsystem. Previous studies that collected data from nurses, directors of nursing, and administrators typically reflected the organizational or meso-level context, rather than the micro-level (Anderson et al., 2003; Barry et al., 2005; Flynn et al., 2010). Such measures of organizational context focused on managerial and operational practices enacted by top management, such as work delegation, nurse participation in organizational decision-making, and plans for reward and career development. Context measures reported by nurses, directors of nursing, and administrators can only be aggregated at the facility level, which reduces sample size and constrains studies to gauge facility-level quality of care as opposed to the more detailed unit-level quality. While studies focusing on organizational or meso-level context contribute to our understanding of the links between organizational context and various quality of care measures such as clinical QIs and inspection deficiencies (Anderson et al., 2003; Barry et al., 2005; Flynn et al., 2010), they overlook important aspects of nursing home care.

The unique attributes of nursing home care, such as a team approach to care, relationship-based care, and care addressing both clinical and psychosocial needs of residents, necessitate a closer examination of context as perceived by care aides. This includes their team dynamics, day-to-day interactions with other staff, relationships with immediate supervisors, and the resources available for their daily practices. Future research could focus on cross-level analysis, simultaneously incorporating both regulated staff and managerial staff-reported organizational context along with care aide-reported unit-level context. This could provide a more comprehensive understanding of their independent and interactive effects on quality of care outcomes (Costa et al., 2013).

The findings of this study may be practically relevant for quality improvement practice and implementation research. Nursing home providers and policy- and regulation-makers may find this study useful when they develop quality improvement interventions targeting modifiable context factors that are sensitive to QIs. For example, providing structural resources on the care unit to facilitate care aides’ ability to consistently access and use knowledge seems to be a promising strategy to improve quality of care in multiple areas. Further, our study reports substantive between-unit variations in organizational context and quality measures. This would support the feasibility to conduct quality improvement interventions at the unit level that identify modifying context elements as areas for improvement and evaluate the success of improvement efforts using unit-level outcome measures (Lanham, 2013; Nakrem, 2015; Nelson et al., 2002).

Our findings emphasize the importance of incorporating unit-level QIs in both research and practical applications. Solely focusing on facility-level QIs may mask the variations that occur across individual care units within a facility. Through ICCs, we identified the sources of variability in unit-level QIs. For some QIs, the variability predominantly arises from differences at the unit level, while for others, it is a mix of differences found at both unit and facility levels. This observation aligns with Norton et al. (2014) who reported that unit-level statistical process control charts presented different changes in the QIs than did the facility-level charts. Future research could delve deeper into why certain QIs have high ICCs while others have low ICCs. For instance, for QIs such as worsened depressive symptoms, unexplained weight loss, and the use of physical restraints—where almost half of the variability can be attributed to between-facility differences—one may question if certain facility-wide policies or procedures influence these outcomes. Conversely, for QIs like pressure ulcers and worsened behavioral symptoms, which exhibit minimal ICCs, it is worth probing if distinct characteristics of individual care units within a facility, which may be overlooked at the facility level, cause intra-facility variations. This insight is important for quality monitoring and improvement, guiding the decision on whether the unit or the facility should be the primary focus for monitoring and intervention targets.

### Limitations

Due to the cross-sectional design, we were unable to establish causal relationships between organizational context and QIs. Additionally, aggregating organizational context or QI data to the unit level may result in a loss of individual-level information. Caution is needed when making inferences about health outcomes for individual residents based on unit-level aggregated data. Although our sample was representative of the target population, the relatively small size of the analytical sample (i.e., the number of care units included for the analysis) might not afford sufficient statistical power; the insignificant associations between certain context variables and QIs could possibly be false negatives.

Furthermore, our findings may only be generalized to urban nursing homes in Alberta, British Columbia, and Manitoba. Finally, although our ACT measures of organizational context captured various contextual elements and drew from the perceptions and experiences of care aides, the context measures do not specifically assess care processes, techniques, and knowledge use associated with a given QI, nor do they comprehensively embrace views from various groups of staff. In addition, while our survey data were collected through in-person interviews—which can improve accuracy over self-administered surveys by addressing outliers and missing information in real-time—it is important to acknowledge potential response biases like recall bias and social desirability bias.

## Conclusions

This study provides a microsystem look at organizational context and quality of care in nursing homes. Targeting modifiable contextual elements and unit-level quality improvement interventions is promising for quality improvement interventions. Further research is needed to understand the mechanisms by which these contextual elements affect quality of care.

## Supplemental Material

Supplemental Material - Organizational Context and Quality Indicators in Nursing Homes: A Microsystem Look Supplemental Material for Organizational Context and Quality Indicators in Nursing Homes: A Microsystem Look by Yinfei Duan, Matthias Hoben, Yuting Song, Stephanie A. Chamberlain, Alba Iaconi, Katharina Choroschun, Shovana Shrestha, Greta G. Cummings, Peter G. Norton, and Carole A. Estabrooks in Journal of Applied Gerontology
