# Supplementary material for: Organizational Context and Quality Indicators in Nursing Homes: A Microsystem Look
Source: J Appl Gerontol. 2023 Sep 5;43(1):13–25. doi: 10.1177/07334648231200110 (PMC10693724; doi:10.1177/07334648231200110)
Supplement: Supplemental Material - Organizational Context and Quality Indicators in Nursing Homes: A Microsystem Look [file sj-pdf-1-jag-10.1177_07334648231200110.pdf]

Supplemental File S1 Risk adjustment process for the computation of risk-adjusted unit-level quality indicators

The TREC program calculated risk-adjusted unit-level quality indicators (QIs) following the technical guide of the risk adjustment methodology developed by the Canadian Institute for Health Information (CIHI).[1] While the CIHI technical guide describes the risk adjustment process for creating facility-level QIs, the same method applies to the calculation of indicator results at other levels, such as corporations, regions, provinces and territories. The TREC program has used this method for the computation of unit-level QIs. The risk adjustment process compares the risk profile of the resident population on an individual care unit with the profile of a standard reference population and then modifies the QI results for that care unit, so it is relative to the standard reference population.

The process for calculating risk-adjusted unit-level QIs involves first determining the appropriate numerator and denominator at the unit level for each QI. Following this, four steps are undertaken: stratification, indirect standardization via logistic regression, direct standardization, and outlier trimming.

Stratification. For each care unit, this step stratifies the unit population (if facility-level QIs were calculated, this would involve stratifying facility population) into three risk groups, or strata: high, medium and low. The risk group for each indicator are based on either a RAI-MDS 2.0 outcome scale (such as the Cognitive Performance Scale, Activities of Daily Living Long Form Scale) or the Case Mix Index (CMI). The observed (unadjusted) QI score for each risk group on the unit is calculated.

Indirect standardization using logistic regression. This step involves calculating an expected QI score for each risk group on the unit using a logistic regression model that adjusts for multiple resident-level risk covariates that are aggregated to the unit level. The parameters for the logistic regression models (one for each risk group) are calculated from the standard reference population and then applied to the data for each risk group from the unit. A performance ratio for each risk group is subsequently calculated by dividing the observed QI score for the risk group by the respective expected QI score. Finally, the adjusted QI score for each risk group is calculated by multiplying the performance ratio by the QI score from the standard reference population.

Direct standardization and creation of a single adjusted QI score. As each unit has its own unique distribution of residents across the three risk groups, this step modifies the adjusted QI scores to treat each unit as though it had the same distribution among the three risk groups (and the same as the standard reference population). The adjusted QI scores for each risk group are combined to create a single adjusted QI score for that unit.

Outlier trimming. The final step of the risk adjustment is to check the distribution of the adjusted QI scores. If the adjusted score for a specific unit is above (or below) the maximum (or minimum) unadjusted QI score across all the units that are being risk-adjusted, the unit's adjusted QI is "trimmed" to within 10% of the standard deviation of the unadjusted QI.

Supplementary Table 1 provides comprehensive details, including the numerator, denominator, stratification variable, covariates used for calculating each risk-adjusted unit-level QI included in this study.

1. Canadian Institute for Health Information (2013). CCRS Quality Indicators Risk Adjustment Methodology.

Supplemental Table S1 Numerators, denominators, stratification variables, covariates used for calculating each risk-adjusted unit-level quality indicator (QI)

QI code	Description	Numerator	Denominator	Stratification variables	Covariates
RES01	% of residents in daily physical restraints	Residents who were physically restrained daily on their target assessment	Residents with valid assessments, excluding comatose residents and those who are quadriplegic	ADL Long Form	None
PRU05	% of residents who had stage 2 to 4 pressure ulcers	Residents who had a pressure ulcer at stage 2 to 4 on their target assessment	Residents with valid assessments	CMI	• RUG Cognitive Impairment<br>• PSI: Subset 1—Diagnoses<br>• More dependence in toileting<br>• Age younger than 65
WGT01	% of residents who had unexplained weight loss	Residents with weight loss documented on their target assessment	Residents with valid assessments, excluding end-of-life residents and those on a planned weight-loss program	CMI	• Age younger than 65
PAN01	% of residents whose pain worsened	Residents with greater pain (higher Pain Scale score) on their target assessment than on their prior assessment	Residents with valid assessments whose pain symptoms could increase (did not have maximum Pain Scale score on prior assessment)	CMI	• Age younger than 65
BEHD4	% of residents whose behavioral symptoms worsened	Residents with more behavioral symptoms present on their target assessment than on their prior assessment	Residents with valid assessments, excluding comatose residents	CPS	• CPS<br>• Motor agitation<br>• Age younger than 65
DEL0X	% of residents with symptoms of delirium	Residents with any of the following conditions:<br>• One or more behavior symptoms	Residents with valid assessments, excluding comatose and end-of	DRS	• Age younger than 65

		that appeared to be different from usual functioning on their target assessment<br>• One or more behavior symptoms that appeared to be different from usual functioning on their prior assessment and present on their target assessment<br>• Not severely cognitively impaired on their target assessment and one or more behavior symptoms present on their target assessment that was not present on their prior assessment	life residents		
ADL01	% of residents with whose late-loss ADL functioning worsened	Residents with worse late-loss ADL self-performance (increased score) on their target assessment than on their prior assessment	Residents whose late-loss ADL score could worsen (did not have maximum score on prior assessment), excluding comatose and end-of life residents	ADL Long Form	• Age younger than 65
FAL02	% of residents who fell in the last 30 days	Residents who had a fall in the last 30 days recorded on their target assessment	Residents with valid assessments	CMI	• Not totally dependent in transferring<br>• Locomotion problem<br>• PSI: Subset 2— Non-Diagnoses<br>• Any wandering<br>• Unsteady gait/cognitive impairment<br>• Age younger than 65
MOD4A	% of residents whose depressive symptoms	Residents with a higher DRS score on their target assessment than on their prior assessment	Residents with valid assessments whose depression symptoms could worsen (did not	CMI	• Age younger than 65

	worsened		have maximum DRS score on prior assessment), excluding comatose residents		
DRG01	% of residents on antipsychotics without a diagnosis of psychosis	Residents who received antipsychotic medication on their target assessment	Residents with valid assessments, excluding those with schizophrenia, Huntington's chorea and hallucinations, and end-of-life residents	CMI	• Motor agitation<br>• Moderate/impaired decision-making problem<br>• Long-term memory problem<br>• CPS<br>• Combination Alzheimer's disease/other dementia<br>• Age younger than 65

Notes. ADL = Activities of Daily Living, CMI=Case Mix Index, CPS = Cognitive Performance Scale, DRS = Depression Rating Scale, RUG = Resident Utility Group

Supplemental Table S2 Associations between quality indicators and Alberta Context Tool (ACT) scales: Results (regression coefficients and 95% CI) from two-level random intercept linear regression

	RES01 (Daily physical restraints)	PRU05 (Stage 2 to 4 pressure ulcers)	WGT01 (Unexplained weight loss)	PAN01 (Worsened pain)	BEHD4 (Worsened behavioral symptoms)
The Alberta Context Tool scales					
Leadership	0.00 (-0.03, 0.04)	0.03 (-0.01, 0.07)	-0.01 (-0.07, 0.05)	0.00 (-0.05, 0.05)	0.05 (-0.01, 0.11)
Culture	-0.01 (-0.05, 0.03)	-0.02 (-0.07, 0.03)	0.01 (-0.06, 0.08)	0.04 (-0.02, 0.1)	0.03 (-0.04, 0.1)
Social capital	0.01 (-0.03, 0.05)	0.03 (-0.02, 0.08)	0.06 (0, 0.13)	-0.01 (-0.07, 0.05)	0.03 (-0.04, 0.1)
Formal interactions	0.01 (-0.01, 0.04)	-0.01 (-0.04, 0.02)	0.00 (-0.05, 0.04)	-0.01 (-0.05, 0.03)	0.00 (-0.04, 0.05)
Informal interactions	0.00 (-0.01, 0.01)	0.01 (0, 0.03)	0.00 (-0.02, 0.02)	0.01 (-0.01, 0.02)	0.00 (-0.02, 0.02)
Structural resources	0.01 (-0.01, 0.02)	0.01 (-0.01, 0.02)	-0.01 (-0.03, 0.01)	0.00 (-0.02, 0.02)	0.00 (-0.03, 0.02)
OS–space	0.00 (-0.02, 0.01)	0.00 (-0.02, 0.01)	-0.01 (-0.03, 0.01)	0.00 (-0.01, 0.02)	-0.01 (-0.03, 0.01)
OS-time	0.00 (-0.03, 0.02)	-0.02 (-0.05, 0.01)	0.01 (-0.03, 0.06)	-0.04* (-0.07, 0.00)	-0.03 (-0.07, 0.01)
Control variables					
Facility size (Ref=Small or Medium)					
Large	0.00 (-0.02, 0.02)	0.02* (0, 0.04)	-0.01 (-0.05, 0.02)	0.04** (0.01, 0.06)	0.00 (-0.02, 0.03)
Ownership model (Ref=Private for profit)					
Public/voluntary non-profit	0.01 (-0.02, 0.03)	0.01 (-0.01, 0.03)	-0.01 (-0.05, 0.02)	0.02 (-0.01, 0.04)	0.02 (-0.01, 0.04)
Count of services/care programs available in the facility	0.00 (-0.01, 0.00)	0.00 (0.00, 0.00)	0.00 (-0.01, 0.01)	0.01* (0.00, 0.01)	0.01* (0.00, 0.01)
Count of quality of improvement activities carried out in the facility	-0.01 (-0.02, 0.00)	0.00 (-0.01, 0.01)	0.02 (0.00, 0.03)	0.01 (-0.01, 0.02)	0.00 (-0.01, 0.01)
Total care hours per resident day	0.00 (-0.01, 0.02)	-0.01 (-0.02, 0.00)	-0.01 (-0.03, 0.01)	-0.02* (-0.04, -0.01)	-0.01 (-0.03, 0.01)
Percent of care hours per resident day provided by care aides (%)	0.05 (-0.05, 0.15)	0.09 (-0.01, 0.19)	0.08 (-0.10, 0.27)	-0.07 (-0.21, 0.07)	0.04 (-0.11, 0.19)
Intercept	0.03 (-0.16, 0.21)	-0.16 (-0.38, 0.06)	-0.25 (-0.58, 0.07)	0.10 (-0.18, 0.37)	-0.24 (-0.57, 0.08)
Random effect					
Variance (Intercept)	0.00 (0.00, 0.00)	0.00 (0.00, 0.00)	0.00 (0.00, 0.01)	0.00 (0.00, 0.00)	0.00 (0.00, 0.00)

Variance (Residual)	0.00 (0.00, 0.00)	0.00 (0.00, 0.00)	0.01 (0.00, 0.01)	0.00 (0.00, 0.01)	0.01 (0.01, 0.01)
ICC	0.42 (0.29, 0.56)	0.03 (0.00, 0.43)	0.40 (0.26, 0.56)	0.19 (0.10, 0.35)	0.04 (0.00, 0.43)
R^2	0.04	0.08	0.04	0.13	0.07
n	260	259	257	260	260

Supplemental Table S1 (continued)

	DEL0X (Symptoms of delirium	ADL01 (Worsened late-loss ADL)	FAL02 (Fall in the last 30 days	MOD4A (Worsened depressive symptoms)	DRG01 (Antipsychotics)
The Alberta Context Tool scales					
Leadership	0.02 (-0.07, 0.10)	-0.04 (-0.12, 0.04)	0.04 (-0.03, 0.12)	0.01 (-0.06, 0.09)	0.03 (-0.07, 0.13)
Culture	-0.03 (-0.12, 0.07)	0.03 (-0.06, 0.12)	0.04 (-0.05, 0.13)	0.05 (-0.04, 0.13)	0.01 (-0.10, 0.13)
Social capital	0.12* (0.03, 0.21)	0.07 (-0.02, 0.15)	0.03 (-0.06, 0.11)	0.1* (0.02, 0.18)	0.02 (-0.09, 0.13)
Formal interactions	-0.03 (-0.09, 0.03)	0.00 (-0.06, 0.05)	-0.04 (-0.09, 0.02)	-0.03 (-0.08, 0.03)	0.05 (-0.03, 0.12)
Informal interactions	0.01 (-0.01, 0.04)	0.00 (-0.03, 0.02)	-0.01 (-0.04, 0.01)	0.00 (-0.02, 0.03)	0.00 (-0.04, 0.03)
Structural resources	0.00 (-0.03, 0.04)	-0.03* (-0.06, 0.00)	-0.01 (-0.04, 0.02)	-0.01 (-0.04, 0.02)	-0.06** (-0.10, -0.02)
OS–space	0.02 (-0.01, 0.04)	-0.01 (-0.04, 0.01)	-0.01 (-0.03, 0.01)	0.01 (-0.02, 0.03)	-0.02 (-0.05, 0.01)
OS-time	-0.02 (-0.08, 0.04)	-0.02 (-0.07, 0.04)	0.00 (-0.05, 0.05)	-0.03 (-0.08, 0.03)	0.04 (-0.03, 0.11)
Control variables					
Facility size (Ref=Small or Medium)					
Large	0.05* (0.00, 0.09)	0.00 (-0.03, 0.04)	-0.01 (-0.04, 0.03)	0.04 (0, 0.09)	0.00 (-0.04, 0.04)
Ownership model (Ref=Private for profit)					
Public/voluntary non-profit	0.03 (-0.01, 0.08)	0.00 (-0.03, 0.04)	0.03 (0.00, 0.07)	0.03 (-0.02, 0.08)	0.00 (-0.04, 0.05)
Count of services/care programs available in the facility	0.00 (-0.01, 0.01)	0.01 (0.00, 0.02)	0.00 (-0.01, 0.00)	0.01 (0.00, 0.02)	0.01* (0.00, 0.02)
Count of quality of improvement activities carried out in the facility	0.00 (-0.02, 0.02)	0.00 (-0.02, 0.02)	0.00 (-0.01, 0.02)	-0.01 (-0.03, 0.01)	0.00 (-0.02, 0.02)
Total care hours per resident day	-0.01 (-0.04, 0.02)	0.04** (0.01, 0.06)	0.02 (-0.01, 0.04)	-0.01 (-0.04, 0.01)	0.03 (0.00, 0.06)
Percent of care hours per resident day provided by care aides (%)	0.02 (-0.22, 0.26)	-0.12 (-0.32, 0.09)	0.14 (-0.06, 0.33)	0.18 (-0.06, 0.41)	-0.1 (-0.35, 0.16)
Intercept	-0.34 (-0.77, 0.09)	0.05 (-0.36, 0.45)	-0.25 (-0.64, 0.14)	-0.51* (-0.91, -0.11)	-0.12 (-0.64, 0.39)
Random effect					
Variance (Intercept)	0.01 (0.00, 0.01)	0.00 (0.00, 0.01)	0.00 (0.00, 0.00)	0.01 (0.00, 0.01)	0.00 (0.00, 0.01)
Variance (Residual)	0.01 (0.01,	0.01 (0.01,	0.01 (0.01,	0.01 (0.01,	0.02 (0.01,

2. Nakagawa, S., & Schielzeth, H. (2013). A general and simple method for obtaining R2 from generalized linear mixed- effects models. Methods in ecology and evolution, 4(2), 133-142.

	0.01)	0.01)	0.01)	0.01)	0.02)
ICC	0.35 (0.23, 0.50)	0.19 (0.08, 0.38)	0.17 (0.07, 0.34)	0.48 (0.34, 0.61)	0.11 (0.03, 0.30)
R^2	0.06	0.08	0.07	0.07	0.08
n	255	256	259	259	256

Notes. For each QI, we used 2-level mixed-effect linear regression to examine the relationship of the QI with eight of the Alberta Context Tool scales, controlling for the clustering of care units nested within the same facility. We also controlled for covariates including facility size, ownership model, services/care programs, quality of improvement activities, total care hours per resident day, percent of care hours per resident day provided by care aides. Marginal R^2 was calculated, which focuses on variance of the outcome explained by fixed factors.[2] We used $Variance_{null}$-$Variance_{model}$/$Variance_{null}$ to calculate marginal R^2. $Variance_{model}$ is the residual variance of the full model that includes fixed effects of predictors and the random effect. $Variance_{null}$ is the residual variance of the null model that includes the intercept and random effect only with the random effect being constrained to be the same as that in the full model. Coefficients and 95% CIs of the covariates are presented in the Supplemental Table S2.

*p<0.05; **p<0.01; ICC=Intra-class correlation; OS: Organizational slack

2. Nakagawa, S., & Schielzeth, H. (2013). A general and simple method for obtaining R2 from generalized linear mixed- effects models. Methods in ecology and evolution, 4(2), 133-142.
